# Maternal pre-pregnancy BMI, gestational weight gain, and the cardiovascular stress response in the adolescent offspring

**DOI:** 10.1016/j.ajpc.2026.101606

**Published:** 2026-04-04

**Authors:** Arwen S.J. Kamphuis, Alexander Hirsch, Ricardo P.J. Budde, Arno A.W. Roest, Vincent W.V. Jaddoe, Romy Gaillard

**Affiliations:** a The Generation R Study Group, Erasmus University Medical Center, Rotterdam, the Netherlands; b Department of Pediatrics, Sophia Children’s Hospital, Erasmus University Medical Center, Rotterdam, the Netherlands; c Department of Cardiology, Cardiovascular Institute, Thorax Center, Erasmus University Medical Center, Rotterdam, the Netherlands; d Department of Radiology and Nuclear Medicine, Erasmus University Medical Center, Rotterdam, the Netherlands; e Department of Pediatrics, Leiden University Medical Center, Leiden, the Netherlands

**Keywords:** Maternal pre-pregnancy BMI, Gestational weight gain, Adolescent, Offspring, Cardiovascular stress, Exercise test, Cardiovascular magnetic resonance

## Abstract

**Background:**

Maternal pre-pregnancy overweight and excessive gestational weight gain (GWG) are important risk factors for cardiovascular disorders in offspring. Underlying mechanisms remain unclear, but may involve structural and functional cardiovascular developmental adaptions. To detect subtle cardiovascular adaptations not present at rest, exercise testing can be used. We examined the associations of maternal pre-pregnancy BMI and GWG with the cardiovascular stress response in adolescent offspring.

**Methods:**

Among 180 mother-adolescent-pairs from a population-based prospective cohort from early-pregnancy onwards, we obtained maternal pre-pregnancy BMI and GWG by physical measurements and questionnaires. At 16 years, offspring performed an isometric handgrip exercise with continuous heart rate and blood pressure (BP) monitoring and cardiovascular MRI.

**Results:**

Maternal pre-pregnancy overweight was associated with higher adolescent peak systolic BP (compared to maternal normal weight: difference: 0.45SDS, 95% confidence interval:0.12–0.78 SDS), but not with systolic BP at rest or recovery. This association was also present across-the-full-range of maternal pre-pregnancy BMI and not explained by birth characteristics or adolescent BMI. No differences were observed in heart rate or diastolic BP response to exercise by maternal pre-pregnancy BMI across-the-full-range, in clinical categories or by GWG. Higher GWG across-the-full-range, but not maternal pre-pregnancy BMI or excessive GWG, was associated with higher left ventricular stroke volume, ejection fraction, and cardiac index during stress (all p-values<0.05). These associations were not explained by birth characteristics or adolescent BMI.

**Conclusions:**

Higher maternal pre-pregnancy BMI and GWG across-the-full-range are associated with higher systolic BP at peak exercise and an altered cardiac response to exercise in adolescent offspring, respectively. Optimizing maternal weight before and during pregnancy may aid in prevention of cardiovascular risk development in offspring.

## Introduction

Maternal pre-pregnancy overweight and obesity are important risk factors for cardiovascular dysfunction in offspring throughout the life course [[1], [2], [3], [4]]. In a record linkage analysis among 37,709 participants, maternal overweight and obesity during pregnancy were associated with increased risks of offspring premature all-cause mortality and hospital admission due to cardiovascular events in adulthood [5]. Next to maternal pre-pregnancy weight, high gestational weight gain (GWG) has also been related to an adverse offspring cardiovascular profile, although associations are less consistent [3,4,6,7]. Underlying mechanisms remain to be established, but may involve altered embryonic and fetal cardiovascular development due to an adverse intra-uterine environment, including cardiomyocyte hypertrophy, and proliferation, and higher sympathetic hyper-reactivity, leading to increased peripheral vascular resistance, hypertension, cardiac remodeling and higher risks of offspring cardiovascular diseases [1,[8], [9], [10], [11], [12]]. Next to these direct mechanisms, shared family-based lifestyle and socio-demographic factors, and offspring concurrent weight status may play a role. Among 4852 children of 6 years of age participating in the Generation R cohort study, we already showed that a higher maternal pre-pregnancy BMI and GWG during early pregnancy were associated with higher systolic blood pressure, left ventricular mass, and aortic root diameter [2,[6], [7], [8]], partly explained by offspring weight.

Most previous studies on offspring cardiovascular outcomes are performed at rest. During physical exercise, which is a stressor for the cardiovascular system, the cardiovascular system needs to adapt adequately to maintain a proper blood flow and oxygen supply. During cardiovascular stress, subtle cardiovascular dysfunction can be revealed which is not apparent at rest [13,14]. An adequate response to exercise is reflected by increased heart rate, blood pressure, and increased stroke volume [15,16]. Suboptimal cardiovascular response to exercise is associated with poorer cardiovascular health in children, and related to the development of cardiovascular events and death during adulthood [[16], [17], [18], [19], [20]]. One experimental study performed in mice investigated whether parental obesity alters the offsprings’ heart rate and mean arterial pressure responses to air-jet-induced stress and observed an enhanced blood pressure response in males born from parents with obesity, independent from current body size [21].

We hypothesized that adolescents born from mothers with overweight or obesity or excessive GWG have an impaired cardiovascular stress response to exercise compared to children of mothers with a healthy weight status, reflected by altered heart rate response, higher blood pressure and decreased cardiac volumes. We examined the associations of maternal pre-pregnancy weight status, and GWG with the offspring cardiovascular stress response measured by cardiovascular magnetic resonance (CMR) at the age of 16 years among a subgroup of 180 adolescents participating in a population-based prospective cohort study from early pregnancy onwards.

## Methods

### Study design and population

**Fig. 1:**
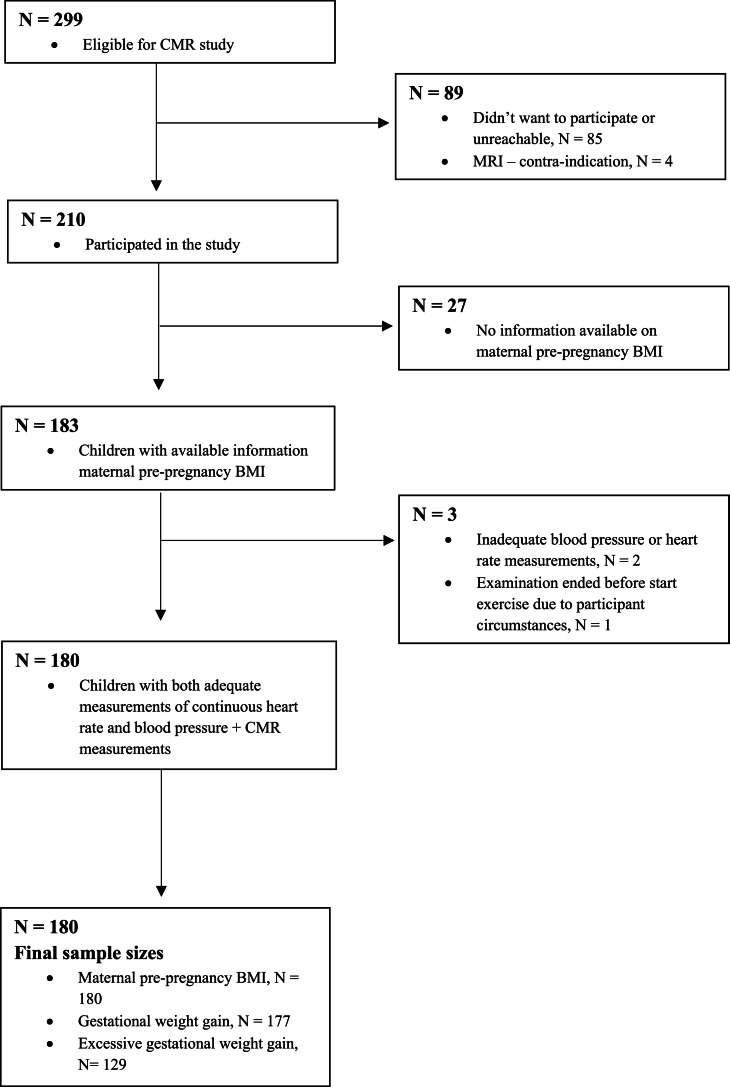
Flowchart of study population.

As described previously, this sub-study was embedded in the Generation R Study, a population-based prospective cohort study from fetal life onwards in Rotterdam, the Netherlands [22,23]. This study was approved by the Medical Ethical Committee of Erasmus MC University Medical Center Rotterdam, The Netherlands. We obtained written informed consent from all parents and participants. In total, 8879 pregnant women were enrolled between 2001 and 2005. A selected subsample of 1184 Dutch women had children with detailed assessments of fetal growth from first trimester onwards, postnatal growth and cardiovascular development until late childhood. We invited a random subgroup from this subsample of 299 adolescents at the age of 16 to participate in a 7-minute cardiovascular exercise test combined with CMR [22,23]. We oversampled adolescents with overweight or obesity, who were born small for their gestational age, or born preterm based on our a-priori hypothesis. From the invited subsample, 210 (70%), adolescents visited the research center for participation in the cardiovascular exercise test [22]. In total, 27 mothers and adolescents with missing data on maternal pre-pregnancy BMI, and 3 adolescents with missing blood pressure data were excluded from analyses, leaving 180 mother-adolescents-pairs available for analysis (Fig. 1).

### Maternal pre-pregnancy BMI and gestational weight gain

As described previously [2,7], we measured maternal weight (in kg) and height (in cm) at enrolment without shoes and heavy clothing. We obtained information on maternal weight just before pregnancy by questionnaire. We calculated pre-pregnancy BMI (kg/m^2^). We categorized maternal pre-pregnancy BMI into two clinical categories: normal weight (BMI<25.0 kg/m^2^) and overweight (BMI ≥ 25.0 kg/m^2^). Total GWG was based on maternal weight measurements in third trimester (timing of visit: median 30.2 weeks of gestation, interquartile-range (IQR) 29.9–30.9), and calculated as third trimester weight minus pre-pregnancy weight. Due to design of the cohort, in a subgroup of N = 129 women, information about maximum weight during pregnancy was assessed by questionnaire 2 months after delivery. We defined excessive GWG in relation to maternal pre-pregnancy weight according to the Institute of Medicine guidelines: for women with underweight, a total weight gain >18 kg, for normal weight >16 kg, for overweight >11.5 kg, and for mothers with obesity >9 kg [24].

### Cardiovascular response during the cardiovascular exercise test

As described previously, we used a sustained isometric handgrip exercise to provoke a cardiovascular stress reaction [22,23]. A detailed description is provided in supplementary text S1. Heart rate and blood pressure were continuously measured by the CareTaker finger cuff system (Empirical Technologies Corporation, Charlottesville, Virginia). We converted continuous measurements into mean measurements of heart rate and blood pressure for predefined time-intervals [25,26]: mean of 5 min rest before the cardiovascular exercise test, means of each minute of the 7-minute exercise, and the mean of each 30 s in the first 2 min after exercise cessation and the mean of the 5th minute after exercise cessation. 3 specific timepoints were defined: rest (mean value of 5 min before start), peak exercise (mean value of the 7th minute of exercise), and recovery (mean value in the 5th minute after exercise).

Cardiac measures were obtained by CMR using a 3T clinical MRI scanner (Discovery MR750w, GE Healthcare, Milwaukee, WI, USA) in resting state and during exercise, as described previously [22,23]. The protocol is described in supplementary text S2. Outcomes of interest were: left ventricular end-diastolic volume and end-systolic volume, stroke volume, ejection fraction, cardiac output, cardiac index, aorta distensibility, and aorta pulse wave velocity. Aorta distensibility and pulse wave velocity of the ascending aorta were calculated as described previously [22].

### Covariates

Information about maternal age, educational level, parity, and smoking during pregnancy was obtained through questionnaires. At the time of the exercise CMR, when the adolescents visited the research center, length and weight were obtained without shoes and heavy clothing.

### Statistical analysis

First, population characteristics in the total group, and between maternal pre-pregnancy BMI categories were assessed using *t*-tests, Mann-Whitney-*U tests*, or Chi-square-tests when applicable. Second, using linear mixed-effects models to account for the correlation between the measurements of each participant, we examined differences in the cardiovascular response of heart rate and blood pressure over time for maternal pre-pregnancy BMI in clinical categories and maternal pre-pregnancy BMI across the full range. A detailed description is provided in supplementary text S3. Third, to conduct a more in-depth analysis of the associations of maternal pre-pregnancy BMI and GWG across the full range and in clinical categories with the cardiovascular response to exercise in adolescents, we used linear regression models with different adjustments for potential confounders and mediators. We focused on heart rate, systolic blood pressure, diastolic blood pressure, and mean arterial pressure at three predefined timepoints: rest, peak exercise, and recovery, and on CMR measurements at rest and peak exercise. To compare effects across outcomes, we constructed standard deviation scores (SDS) for maternal pre-pregnancy BMI, GWG and all adolescent cardiovascular outcomes. As previously described [14], we standardized all CMR outcomes, except LVEF, to account for different body surface area (BSA). We constructed 5 models: 1) a basic model, 2) a confounder model, which we considered our main model, adjusted for maternal age, education level, parity and smoking during pregnancy. For the GWG analyses, we constructed an additional confounder model which also included maternal pre-pregnancy BMI; 3) a birth model which was the confounder model additionally adjusted for birth weight and gestational age at birth; 4) a childhood model, which was the confounder model additionally adjusted for adolescent BMI; 5) a fully adjusted which included all maternal confounders and potential mediators. We only performed models 3 to 5 for those outcomes, for which we observed significant associations in the confounder model. A detailed description of the procedures used to incorporate the covariates, is provided in supplementary text S4. As all outcomes are highly correlated, we did not correct for multiple testing [27]. Given the relatively small sample size and the number of tests performed, this study needs to be considered as hypothesis generating. Missing values of covariates and maximum GWG, were imputed using multiple imputation via the R Package ‘MICE’ [28], version 3.16.0, using 25 imputed datasets. All statistical analyses were performed using R statistical software, V4.4.1.

## Results

### Participant characteristics

130 (72%) mothers had a normal pre-pregnancy weight, and 50 (28%) had pre-pregnancy overweight. Mothers with overweight had a lower educational level, more often developed excessive GWG, and gestational hypertension compared to mothers with a normal weight (all p-values <0.05). No differences in birth characteristics or other pregnancy complications were present between maternal BMI categories. Adolescents with a mother with overweight were heavier, and had a higher adolescent BMI, as compared to adolescents born to a mother with normal weight (p-values <0.05, Table 1).

**Table 1:** General characteristics of the study population.

	Total	Maternal Normal weight	Maternal Overweight	P-value
	N = 180	N = 130	N = 50	
Maternal characteristics				
Maternal age in years, mean (±SD)	32.1 (±3.8)	32.1 (±3.8)	32.1 (±3.9)	0.992
Educational level, n (%) None, primary or secondary Higher	54 (30.0%) 126 (70.0%)	26 (20.0%) 104 (80.0%)	28 (56.0%) 22 (44.0%)	<0.001
Parity, n (%) Nullipara Multipara	113 (62.3%) 67 (37.2%)	84 (64.6%) 46 (35.4%)	29 (58.0%) 21 (42.0%)	0.516
Smoking, n (%) Never during pregnancy Until pregnancy was known Continued during pregnancy	131 (77.5%) 18 (10.7%) 20 (11.8%)	96 (78.7%) 14 (11.5%) 12 (9.8%)	35 (74.5%) 4 (8.5%) 8 (17.0%)	0.399
Height in centimeters, mean (±SD)	171.9 (±6.8)	172.4 (±6.6)	170.7 (±7.2)	0.127
Weight in kilograms, median (IQR)	68.0 (60.8 −76.0)	63.0 (59.0 - 69.0)	81.5 (76.0 - 88.5)	<0.001
Pre-pregnancy BMI in kilograms/m^2^, median (IQR)	22.3 (20.7 - 25.4)	21.4 (20.3 - 22.8)	28.0 (26.4 - 29.5)	<0.001
Gestational weight gain in kilograms, median (IQR)	10.0 (7.0 −13.0)	10.5 (7.0 - 12.8)	9.0 (7.0 - 13.0)	0.639
Excessive gestational weight gain, n (%) Normal Excessive	65 (50.4%) 64 (49.6%)	56 (59.6%) 38 (40.4%)	9 (25.7%) 26 (74.3%)	0.001
Gestational hypertensive disorders, n (%) Gestational hypertension Pre-eclampsia None	16 (9.0%) 3 (1.7%) 155 (87.6%)	5 (3.9%) 1 (0.8%) 121 (95.3%)	11 (22.0%) 2 (4.0%) 34 (68.0%)	<0.001
Gestational diabetes, n (%) Yes No	1 (0.6%) 178 (99.4%)	0 (0.0%) 129 100%)	1 (2.0%) 49 (98%)	0.622
				
Child characteristics - birth				
Gestational age at birth in weeks, median (IQR)	40.3 (39.3 - 41.0)	40.3 (39.4 - 41.1)	40.4 (39.2 - 40.9)	0.903
Sex, N (%) Boys Girls	96 (53.3%) 84 (46.7%)	71 (54.6%) 59 (45.4%)	25 (50.0%) 25 (50.0%)	0.697
Birth weight in grams, mean (±SD)	3455 (±556)	3417 (±585)	3550 (±465)	0.151
Child characteristics – moment of measurement				
Age in years, median (IQR)	16.1 (15.6 - 16.9)	16.1 (15.7 - 16.9)	16.1 (15.6 - 16.8)	0.668
Weight in kilograms, median (IQR)	64.5 (57.8 - 74.0)	62.0 (56.0 - 69.8)	71.0 (64.2 - 83.8)	<0.001
Height in cm, mean (±SD)	175.4 (±8.3)	175.6 (±8.3)	175.1 (±8.6)	0.755
BMI, in kg/m^2,^ median (IQR)	20.7 (19.1 - 23.4)	20.0 (18.7 - 22.2)	22.5 (21.1 - 26.2)	<0.001
Resting heart rate in bpm, mean (±SD)	66.0 (±8.4)	66.0 (±8.3)	66.1 (±9.0)	0.940
Resting systolic blood pressure in mmHg, mean (±SD)	116.5 (±11.7)	115.5 (±11.7)	119.1 (±11.4)	0.059
Resting diastolic blood pressure in mmHg, mean (±SD)	69.1 (±8.3)	68.5 (±8.3)	70.8 (±8.1)	0.092
Resting mean arterial pressure in mmHg, mean (±SD)	83.8 (±8.6)	82.7 (±8.4)	86.6 (±8.4)	0.006
Mean contraction during exercise in kgf/m^2^, median (IQR)	1993 (1748 - 2317)	1997 (1770 - 2329)	1988 (1722 - 2279)	0.370
Maximal voluntary contraction in kgf/m^2^, median (IQR)	5941 (5083 - 6982)	5955 (5070 - 7226)	5874 (5153 - 6784)	0.550
Smoking, N (%) Yes No	13 (7.3%) 164 (92.7%)	11 (8.7%) 116 (91.3%)	2 (4.0%) 48 (96.0%)	0.453
CMR measurements at rest				
Left ventricular mass *(*gram*)*, median (IQR)	86 (76 – 102)	85.7 (77 – 102)	85 (75 – 103)	0.819
Left ventricular end-diastolic volume *(*ml*),* mean (±SD)	171 (±26)	169 (±26)	177 (±25)	0.078
Left ventricular end-systolic volume *(*ml*)* mean (±SD)	79 (±15)	78 (±15)	82 (±16)	0.106
Left ventricular stroke volume*(*ml/ heart beat*)* median (IQR*)*	93 (82 – 103)	91 (80 – 103)	98 (85 – 104)	0.194
Left ventricular ejection fraction *(*%*)* mean (±SD)	54 (±4)	55 (±4)	54 (±4)	0.113
Cardiac output *(*L/min*)* mean (±SD*)*	6 (±1)	6 (±1)	6 (±1)	0.268
Aortic distensibility *(*10^–3^ mmHg*)* median (IQR)	11 (9 – 13)	11 (9 – 12)	10 (9 – 13)	0.887
Aortic pulse wave velocity *(*m/s*)* median (IQR)	4 (3 – 5)	4 (3 – 5)	4 (3 – 5)	0.854

Values are means (standard deviations, SD) or medians (inter quartile range, IQR) or observed numbers (valid percentages, %). Valid percentages represent the percentage of only non-missing cases in each category of categorical variable.

*if continuous variable and normally distributed, one-way ANOVA was used, if continuous variable and not normally-distributed Kruskal Wallis test was used. For categorical variables chi-square or Fisher’s exact test was used.

Significant P-values are reported as <0.05 or <0.01 accordingly.

All CMR measurements are at rest.

BMI: Body Mass Index, Bpm: beats per minute, CMR: Cardiovascular Magnetic Resonance, IQR: Inter Quartile Range, SD: Standard Deviation.

Normal weight: BMI <25 kg/m2, overweight: BMI ≥ 25 kg/m2.

### Heart rate and blood pressure response

**Fig. 2:**
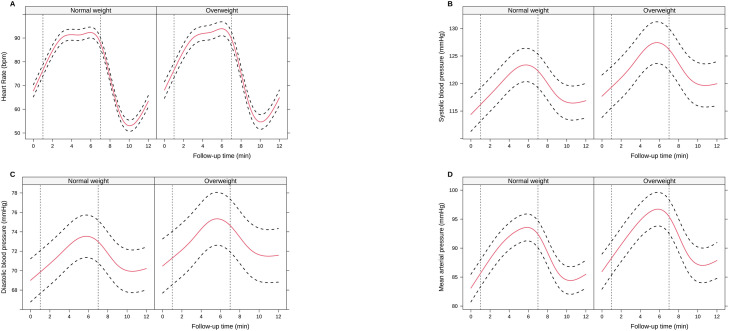
**A-D: Cardiovascular response of heart rate and blood pressure values over time, difference between maternal pre-pregnancy normal weight and overweight**. Adjusted for smoking during pregnancy, maternal age, educational level and parity (confounders set to the mean or reference category, respectively) Complete cases only, total n = 169, normal weight: n = 122, overweight n = 47. Estimated from linear mixed-effects models with natural cubic splines, two internal-knots. Likelihood ratio test to assess differences between the longitudinal patterns among different groups: heart rate: P-value: 0.90, systolic blood pressure: P-value: 0.06, diastolic blood pressure: 0.42, mean arterial blood pressure: P-value: 0.15. Linear mixed models with maternal BMI continuously (SDS), adjusted for smoking during pregnancy, maternal age, educational level and parity: (figures not shown, heart rate p-value: 0.84, systolic blood pressure: p-value: 0.15, diastolic blood pressure: p-value: 0.60, mean arterial pressure: p-value: 0.33).

The longitudinal response before, during, and after exercise of heart rate, systolic blood pressure, diastolic blood pressure, and mean arterial pressure of adolescents by maternal pre-pregnancy BMI status is shown in Fig. 2A-D. All adolescents showed a clear peak in both heart rate and systolic, diastolic, and mean arterial blood pressure after exercise started. After cessation of the exercise, all adolescents showed a decline in heart rate and systolic, diastolic, and mean arterial blood pressure, before stabilizing. Especially, heart rate dropped below baseline at rest, and thereafter stabilized. Stratified analysis showed that among adolescents born from mothers with pre-pregnancy overweight, as compared to adolescents born from mothers with a normal pre-pregnancy weight, systolic blood pressure tended to rise more during exercise and tended to remain higher during recovery. No differences were present for heart rate, diastolic blood pressure, or mean arterial pressure. No differences were observed in the response to exercise by maternal pre-pregnancy BMI across the full range for heart rate, systolic, diastolic, or mean arterial blood pressure.

Table 2 shows the associations of maternal pre-pregnancy BMI and GWG, across the full range and in clinical categories, with adolescent heart rate, systolic and diastolic blood pressure at rest, peak exercise, and recovery, and the role of potential confounders and mediators. In the confounder model, maternal pre-pregnancy overweight was associated with a higher adolescent peak systolic blood pressure (difference: 0.45 SDS, corresponding with 6 mmHg, 95% confidence interval (CI): 0.12–0.78 SDS, 2–10 mmHg) compared to maternal normal weight, but not with systolic blood pressure at rest or recovery. This association was also present across the full range of maternal pre-pregnancy BMI (difference 0.15 SDS per SDS increase in maternal pre-pregnancy BMI, corresponding with 2 mmHg, 95% CI: 0.01–0.29 SDS, 0–4 mmHg). Similar associations were present in the basic model. Additional adjustment for birth characteristics or adolescent BMI did not explain these associations. No associations of maternal pre-pregnancy BMI across the full range or in categories with heart rate or diastolic blood pressure were observed in the confounder model. No associations of GWG with adolescent heart rate, systolic, or diastolic blood pressure were present (Table 2). In line with peak systolic blood pressure, a higher maternal pre-pregnancy BMI across the full range and maternal pre-pregnancy overweight were also associated with higher peak mean arterial pressure (difference 0.15 SDS per SDS increase in maternal pre-pregnancy BMI, corresponding with 2 mmHg, 95% CI: 0.01–0.30 SDS, 0–3 mmHg, and 0.49 SDS, corresponding with 5 mmHg, 95%CI, 0.15 - 0.82 SDS, 2–8 mmHg, respectively), but not at rest or recovery (Supplemental Table S6).

**Table 2:** Maternal BMI, and gestational weight gain and cardiovascular outcomes at rest, peak exercise and recovery.

Cardiovascular outcomes:	Heart rate (SDS) *Rest* N = 177	Heart rate (SDS) *Peak* N = 176	Heart rate (SDS) *Recovery* N = 177	SBP (SDS) *Rest* N = 180	SBP (SDS) *Peak* N = 176	SBP (SDS) *Recovery* N = 178	DBP (SDS) *Rest* N = 179	DBP (SDS) *Peak* N = 174	DPB (SDS) *Recovery* N = 177
**Basic model**									

Normal weight *N= 130*	Ref	Ref	Ref	Ref	Ref	Ref	Ref	Ref	Ref
Overweight *N= 50*	0.01 (-0.31 - 0.34)	0.16 (-0.16 - 0.48)	0.08 (-0.25 - 0.40)	0.31 (-0.01 - 0.63)	**0.40 (0.08 - 0.72)**+	0.27 (-0.06 - 0.59)	0.28 (-0.05 - 0.60)	**0.35 (0.02 - 0.67)**+	0.26 (-0.06 - 0.58)
BMI in SDS *N=180*	0.00 (-0.14 - 0.14)	0.08 (-0.06 -0.21)	0.01 (-0.13 - 0.15)	0.13 (-0.01 - 0.27)	**0.15 (0.01 - 0.28)**+	0.12 (-0.02 - 0.25)	0.09 (-0.05 - 0.23)	0.11 (-0.02 - 0.25)	0.09 (-0.05 - 0.22)

**Confounder model**°									

Normal weight *N= 130*	Ref	Ref	Ref	Ref	Ref	Ref	Ref	Ref	Ref
Overweight *N= 50*	-0.02 (-0.38 - 0.33)	0.18 (-0.17 - 0.53)	0.05 (-0.30 - 0.41)	0.23 (-0.11 - 0.56)	**0.45 (0.12 - 0.78)**+	0.21 (-0.13 - 0.55)	0.16 (-0.19 - 0.51)	0.30 (-0.05 - 0.64)	0.17 (-0.18 - 0.52)
BMI in SDS *N=180*	-0.01 (-0.16 - 0.14)	0.09 (-0.06 - 0.24)	0.00 (-0.15 - 0.15)	0.09 (-0.06 - 0.23)	**0.15 (0.01 - 0.29)**+	0.08 (-0.06 - 0.23)	0.05 (-0.09 - 0.20)	0.09 (-0.06 - 0.24)	0.06 (-0.09 - 0.20)

**Mediator model – Birth characteristics**¶									

Normal weight *N= 130*					Ref				
Overweight *N= 50*					**0.42 (0.09 – 0.75)**+				
BMI in SDS *N=180*					0.14 (-0.00 – 0.28)				

**Mediator model - BMI child**§									

Normal weight *N= 130*					Ref				
Overweight *N= 50*					**0.48 (0.12 – 0.84)**+				
BMI in SDS *N=180*					**0.15 (0.00 – 0.30)**+				

**Fully adjusted model**°°									

Normal weight *N= 130*	Ref	Ref	Ref	Ref	Ref	Ref	Ref	Ref	Ref
Overweight *N= 50*					**0.48 (0.13 - 0.83)**+				
BMI in SDS *N=180*					**0.16 (0.00 – 0.31)**+				

**Gestational weight gain – Basic model**									

Normal *N = 95*	Ref	Ref	Ref	Ref	Ref	Ref	Ref	Ref	Ref
Excessive *N = 85*	-0.12 (-0.43 - 0.19)	-0.03 (-0.34 - 0.27)	-0.11 (-0.42 - 0.20)	0.13 (-0.19 - 0.44)	0.12 (-0.19 - 0.44)	0.13 (-0.19 - 0.45)	0.01 (-0.31 - 0.33)	-0.02 (-0.35 - 0.31)	0.03 (-0.29 - 0.36)

Gestational weight gain in SDS *N = 177*	-0.05 (-0.21 - 0.10)	0.02 (-0.14 - 0.17)	-0.09 (-0.25 - 0.06)	0.05 (-0.10 - 0.21)	-0.01 (-0.17 - 0.14)	0.02 (-0.13 - 0.18)	0.02 (-0.14 - 0.18)	-0.03 (-0.19 - 0.13)	0.01 (-0.15 - 0.17)

**Gestational weight gain – Confounder model**°									
Normal *N = 95*	Ref	Ref	Ref	Ref	Ref	Ref	Ref	Ref	Ref
Excessive *N = 85*	-0.15 (-0.46 - 0.17)	-0.04 (-0.36 - 0.28)	-0.14 (-0.46 - 0.18)	0.03 (-0.29 - 0.35)	0.06 (-0.25 - 0.38)	0.05 (-0.28 - 0.37)	-0.09 (-0.42 - 0.24)	-0.10 (-0.44 - 0.24)	-0.06 (-0.40 – 0.27)
Gestational weight gain in SDS N = 177	-0.07 (-0.23 - 0.10)	0.02 (-0.14 - 0.19)	-0.11 (-0.28 - 0.06)	0.04 (-0.12 - 0.20)	-0.04 (-0.20 - 0.12)	0.00 (-0.16 - 0.16)	0.01 (-0.16 - 0.17)	-0.04 (-0.20 - 0.12)	-0.01 (-0.18 - 0.15)

Values represent regression coefficients (95% confidence interval) from linear regression models that reflect differences in cardiovascular measures in SDS.

SBP: Systolic blood pressure, DBP: Diastolic blood pressure, SDS: standard deviation score.

° Confounder model: adjusted for maternal smoking during pregnancy, parity, educational level and age.

¶ Mediator model – birth characteristics: includes maternal confounders and additionally adjusted for birth weight and gestational age at birth.

§ Mediator model - BMI child at time CMR: includes maternal confounders and additionally adjusted for adolescent BMI at MRI.

+ P < 0.05.

°° Fully adjusted model: includes all maternal confounders and additionally adjusted for birth characteristics and adolescent BMI at MRI.

### Cardiovascular magnetic resonance

In the confounder model, we observed no associations of maternal pre-pregnancy BMI across the full range or in clinical categories with CMR measurements. In the basic model, a higher maternal pre-pregnancy BMI was associated with lower left ventricular end-diastolic volume at rest, lower left ventricular stroke volume at rest and stress, and with lower left ventricular cardiac index at rest, but these were fully explained by confounding (Table 3).

**Table 3:** Maternal BMI and CMR measurements at rest and stress.

	Left ventricular end-diastolic volume *Rest* N = 175	Left ventricular end-diastolic volumes *Stress* N = 156	Left ventricular end-systolic volume *Rest* N = 177	Left ventricular end-systolic volume *Stress* N = 157	Left ventricular stroke volume *Rest* N = 176	Left ventricular stroke volume *Stress* N = 157	Left ventricular ejection fraction *Rest* N = 179	Left ventricular ejection fraction *Stress* N = 159	Left ventricular cardiac index *Rest* N = 175	Left ventricular cardiac index *Stress* N = 154
Basic model										
Normal weight *N= 130* Overweight *N= 50* BMI in SDS *N = 180*	Ref −0.24 (−0.56 - 0.09) **−0.17 (−0.31 -** **−0.03)**+	Ref −0.23 (−0.57- 0.11) −0.11 (−0.26 - 0.03)	Ref −0.12 (−0.44 - 0.20) −0.10 (−0.24 - 0.05)	Ref −0.05 (−0.39 - 0.29) −0.02 (−0.17 - 0.13)	Ref **−0.33 (−0.66 - −0.00)**+ **−0.17 (−0.30 - −0.03)**+	Ref **−0.33 (−0.66 - −0.00)**+ **−0.18 (−0.32 - −0.04)**+	Ref −0.15 (−0.48 - 0.18) −0.05 (−0.19 - 0.09)	Ref −0.11 (−0.44 - 0.22) −0.10 (−0.24 - 0.05)	Ref −0.30 (−0.62 - 0.03) **−0.15 (−0.28 - −0.01)**+	Ref −0.20 (−0.54 - 0.14) −0.10 (−0.24 - 0.05)
Confounder model°										
Normal weight *N= 130* Overweight *N= 50* BMI in SDS *N = 180*	Ref −0.10 (−0.44 - 0.25) −0.13 (−0.28 - 0.03)	Ref −0.05 (−0.40 - 0.30) −0.07 (−0.22 - 0.09)	Ref 0.01 (−0.34 - 0.35) −0.05 (−0.20 - 0.10)	Ref 0.07 (−0.29 - 0.43) 0.01 (−0.15 - 0.17)	Ref −0.22 (−0.58 - 0.13) −0.14 (−0.29 - 0.01)	Ref −0.17 (−0.51 - 0.17) −0.14 (−0.29 - 0.01)	Ref −0.18 (−0.53 −0.17) −0.09 (−0.24 - 0.06)	Ref −0.09 (−0.45 - 0.27) −0.10 (−0.25 - 0.06)	Ref −0.23 (−0.59 −0.12) −0.13 (−0.28 - 0.02)	Ref −0.02 (−0.38 - 0.33) −0.04 (−0.20 - 0.11)
Gestational weight gain – Basic model										
Normal *N = 95* Excessive *N = 85* Gestational weight gain in SDS *N = 177*	Ref 0.09 (−0.22 - 0.40) 0.08 (−0.07 - 0.24)	Ref 0.01 (−0.32 - 0.34) 0.06 (−0.11 - 0.24)	Ref 0.06 (−0.25 - 0.36) 0.05 (−0.10 - 0.20)	Ref 0.07 (−0.25 - 0.39) −0.02 (−0.20 - 0.15)	Ref 0.03 (−0.30 - 0.36) 0.10 (−0.06 - 0.26)	Ref 0.00 (−0.33 - 0.33) 0.15 (−0.02 - 0.32)	Ref 0.09 (−0.23 - 0.41) 0.03 (−0.13 - 0.19)	Ref 0.12 (−0.19 - 0.44) 0.15 (−0.02 - 0.32)	Ref −0.03 (−0.37 - 0.30) 0.05 (−0.11 - 0.20)	Ref −0.01 (−0.35 - 0.33) 0.13 (−0.04 - 0.31)
Gestational weight gain – Confounder model°										
Normal *N = 95* Excessive *N = 85* Gestational weight gain in SDS *N = 177*	Ref 0.19 (−0.14 - 0.51) 0.12 (−0.04 - 0.28)	Ref 0.11 (−0.22 - 0.43) 0.11 (−0.07 - 0.29)	Ref 0.12 (−0.20 - 0.43) 0.06 (−0.10 - 0.22)	Ref 0.12 (−0.20 - 0.45) −0.01 (−0.19 - 0.17)	Ref 0.14 (−0.20 - 0.48) **0.17 (0.00 - 0.33)**+	Ref 0.10 (−0.23 - 0.42) **0.22 (0.05 - 0.39)**+	Ref 0.12 (−0.21 - 0.45) 0.09 (−0.08 - 0.25)	Ref 0.13 (−0.20 - 0.46) **0.18 (0.00 -** **0.36)**+	Ref 0.04 (−0.31 - 0.39) 0.09 (−0.08 - 0.25)	Ref 0.08 (−0.26 - 0.42) **0.21 (0.03 -** **0.38)**+
Gestational weight gain – Maternal BMI°°										
Normal *N = 95* Excessive *N = 85* Gestational weight gain in SDS *N = 177*					Ref 0.18 (−0.15 – 0.52) 0.14 (−0.03 – 0.31)	Ref 0.15 (−0.18 – 0.47) **0.19 (0.02 – 0.36)**+		Ref 0.17 (−0.16 – 0.50) 0.17 (−0.02 – 0.35)		Ref 0.10 (−0.25 – 0.44) **0.21 (0.03 – 0.38)**+
Gestational weight gain – Birth characteristics¶										
Normal *N = 95* Excessive *N = 85* Gestational weight gain in SDS *N = 177*					Ref 0.12 (−0.23 - 0.46) 0.14 (−0.03 - 0.31)	Ref 0.13 (−0.22 - 0.48) **0.24 (0.06 -** **0.41)**+		Ref 0.15 (−0.20 - 0.50) **0.19 (0.01 - 0.38)**+		Ref 0.09 (−0.27 - 0.45) **0.22 (0.05 - 0.40)**+
Gestational weight gain – BMI child§										
Normal *N = 95* Excessive *N = 85* Gestational weight gain in SDS *N = 177*					Ref 0.21 (−0.11 – 0.53) **0.18 (0.03 – 0.34)**+	Ref 0.18 (−0.12 – 0.48) **0.23 (0.07 – 0.38)**+		Ref 0.14 (−0.19 – 0.47) **0.18 (0.00 – 0.36)**+		Ref 0.12 (−0.22 – 0.45) **0.21 (0.04 – 0.38)**+
Gestational weight gain – Fully adjusted model°°°										
Normal *N = 95* Excessive *N = 85* Gestational weight gain in SDS *N = 177*					Ref 0.16 (−0.15 – 0.47) 0.13 (−0.03 – 0.29)	Ref 0.18 (−0.14 – 0.50) **0.23 (0.06 – 0.40)**+		Ref 0.18 (−0.17 – 0.53) 0.18 (−0.01 – 0.37)		Ref 0.10 (−0.25 – 0.46) **0.23 (0.05 – 0.41)**+

Values represent regression coefficients (95% confidence interval) from linear regression models that reflect differences in BMI per SDS in CMR measures, outcomes both in rest and during stress. All CMR measurements were transformed to BSA-corrected SDS. For LVEF we did not create a BSA-adjusted SDS, but standardized this measure as (observed value mean)/standard deviation.

° °Confounder model: adjusted for maternal smoking during pregnancy, parity, educational level and age.

° ° °Confounder model – maternal BMI: adjusted for maternal smoking during pregnancy, parity, educational level, age and maternal prepregnancy BMI.

¶ Mediator model – birth characteristics: includes maternal smoking during pregnancy, parity, educational level, age and additionally adjusted for birth weight and gestational age at birth.

§ Mediator model - BMI child at time CMR: includes maternal smoking during pregnancy, parity, educational level, age and additionally adjusted for adolescent BMI at MRI. °°°Fully adjusted model - includes maternal smoking during pregnancy, parity, educational level, age and maternal prepregnancy BMI and additionally adjusted for birth characteristics and adolescent BMI at MRI.

+ P-value< 0.05. SDS: standard deviation score.

In the confounder model, a higher GWG across the full range was associated with higher left ventricular stroke volume in rest and during stress (differences: (0.17 SDS, 95% CI 0.00–0.33 and 0.22 SDS, 95% CI, 0.05–0.39), with higher left ventricular ejection fraction (0.18 SDS, 95% CI, 0.00–0.36) and higher left ventricular cardiac index during stress (0.21 SDS, 95% CI 0.03–0.38). Except for left ventricular stroke volume at rest, additional adjustment for maternal pre-pregnancy BMI, birth characteristics or adolescent BMI did not explain these associations. No significant associations of excessive GWG compared to normal GWG with these adolescent CMR measurements were observed, but tendencies went in similar direction. No associations were observed for maternal pre-pregnancy BMI or GWG across the full range and in clinical categories with aorta distensibility or pulse wave velocity (Supplemental Table S7).

## Discussion

Higher maternal pre-pregnancy BMI across the full range and in clinical categories, was associated with higher systolic blood pressure at peak exercise during an isometric cardiovascular stress test in offspring aged 16 years, but not with systolic blood pressure at rest or recovery, heart rate, diastolic blood pressure, or cardiac response. Higher maternal GWG across the full range, but not in clinical categories, was associated with an altered cardiac response to exercise in adolescent offspring, but not with heart or blood pressure. These associations were not explained by birth characteristics or adolescent BMI.

### Interpretation of the main findings

Previous studies have suggested that maternal pre-pregnancy overweight, and to a lesser extent excessive GWG, are associated with cardiovascular dysfunction in offspring, including hypertension, and higher risks for hospital admissions for cardiovascular events. These effects are not limited to the extremes of maternal overweight and excessive gestational weight, but rather present across the full range of maternal pre-pregnancy BMI and GWG [1,2,6,7,29,30].

We used an isometric handgrip exercise to induce a cardiovascular stress response to reveal subtle cardiac adaptations that may not be apparent at rest. Maternal pre-pregnancy overweight has been associated with increased heart rate and blood pressure in offspring, of which systolic blood pressure is most affected, but have mainly been studied at rest [2,31]. Multiple animal studies have shown that offspring of mothers with obesity or on a high-fat diet compared to healthy controls, developed a higher blood pressure at rest and in response to restraint and air-jet induced stress [21,29,32,33]. Effects on cardiac development are less well-studied and findings are inconsistent. A study among 91 infants using echocardiography at rest, observed a thicker left ventricular posterior wall at birth in infants born to mothers with overweight during pregnancy and larger left ventricular end-diastolic and stroke volumes at 1 year of age in children born to maternal pre-pregnancy overweight compared to children of mothers with a healthy weight [34]. A study compared 56 neonates born to mothers with healthy BMI to 31 neonates born to mothers with obesity in first trimester, and observed that maternal obesity was associated with increased heart rate, decreased left ventricular end-diastolic volume and stroke volume on CMR at rest, 72 hours after birth [31]. A recent meta-analysis among 13 studies [35], observed that fetuses of pregnant mothers with obesity compared to mothers with a normal weight had lower left ventricular strain which persisted in infancy. There was no evidence for differences in left ventricular stroke volume between neonates born to women with and without obesity. Few studies have been performed in later childhood. Previously, we have shown among 4852 children of 6 years of age that higher maternal pre-pregnancy BMI was associated with higher systolic blood pressure, left ventricular mass, and aortic root diameter. The associations with cardiac outcomes were fully explained by offspring’s concurrent weight status [2,[6], [7], [8]].

Partly in line with these previous studies we showed that maternal pre-pregnancy overweight was associated with higher adolescent systolic blood pressure during exercise, as compared to maternal pre-pregnancy normal weight, but not with systolic blood pressure at rest or recovery, or with heart rate or diastolic blood pressure. This association was already present across the full range of maternal pre-pregnancy BMI and independent from birth characteristics and adolescent BMI. Increased systolic blood pressure during exercise in adult populations has been associated with the development of hypertension in later life [36,37]. As we had a relatively healthy sub-population and a relatively small number of participants, associations with systolic blood pressure at rest or recovery may be more difficult to detect than systolic blood pressure at peak level during exercise. We found no associations of maternal pre-pregnancy BMI with adolescent heart rate, diastolic blood pressure, or CMR measurements. We used an isometric handgrip exercise protocol, and while it significantly increases heart rate, blood pressure, and cardiac volumes, as compared to treadmill or bicycle exercise, it cannot be performed to maximum exertion [16,22]. Possibly, small differences in cardiac adaptations may not be revealed at this level of exercise. It is also possible that the effects are mainly present in children born to mothers with extreme overweight, and that no differences were observed due to our relatively healthy cohort. 28% of our maternal population was living with overweight, with a median maternal pre-pregnancy BMI of 28.0 and an IQR up to 29.5, indicating that there are only a few cases with severe maternal pre-pregnancy obesity. Alternatively, we are one of the few studies that measured cardiac outcomes at adolescent age. Possibly left ventricular morphologic changes due to maternal obesity during pregnancy are mainly present in fetuses, neonates, and infants, and may become less apparent at older ages. Underlying susceptibility to future cardiovascular risk in adulthood may stem from increased sensitivity of the cardiovascular system to secondary insults, such as stress and overweight, which may manifest only after these secondary insults have occurred [1]. Thus, a higher maternal pre-pregnancy BMI was across the full range associated with higher offspring systolic blood pressure during exercise, but not with heart rate, diastolic blood pressure or cardiac response to exercise. Further studies are needed to investigate the long-term impact of higher maternal pre-pregnancy BMI on the cardiovascular stress response.

Next to maternal pre-pregnancy BMI, GWG in pregnancy may influence offspring cardiovascular outcomes, although findings are less strong compared to maternal pre-pregnancy BMI. Two studies among 10,883 participants aged 17 years and 1400 adults aged 32, observed no effect of GWG on offspring systolic or diastolic blood pressure at rest, already in the basic model [38], and after adjustment for birth characteristics, socioeconomic status, and offspring characteristics [30]. Among 3781 adults aged over 50 years, higher maternal GWG was not associated with cardiovascular diseases [39]. In contrast, a study conducted among 2432 young adults aged 21 years, observed a modest effect of higher GWG with higher systolic blood pressure at rest. Although they adjusted for maternal confounding factors, they did not correct for the potential mediating effect of adolescent BMI [40]. Largely in line with these previous studies, we observed no association of maternal GWG with adolescent heart rate or blood pressure response to exercise.

Few studies examined the associations of GWG with CMR outcomes. We observed that higher maternal GWG was across the full range associated with higher left ventricular stroke volume, ejection fraction and cardiac index during peak exercise, which was not explained by birth characteristics or current adolescent BMI. No associations were observed for these CMR measures at rest or for excessive GWG compared to normal GWG with CMR outcomes. This might be related to assessment of excessive GWG in our cohort. Excessive GWG was only available in a subgroup of N = 129 women and obtained by questionnaire 2 months postnatally. Especially in case of higher weight gain, self-reported weight gain tends to be underestimated [7,41]. As we used multiple imputation for missing values in excessive gestational weight gain to not reduce sample size, this might have reduced variation in excessive GWG. These methodological limitations might have led to less power to detect associations of excessive GWG with offspring outcomes as compared to total GWG, which was based on measurements obtained during third trimester visits at the research centre and available for the full sample. Within the Generation R cohort, we have previously shown that, independent from current weight and length, GWG across the full range was associated with higher left ventricular mass in infancy [42], and GWG in early pregnancy was associated with higher left ventricular mass, left ventricular mass index and aortic root diameter at the age of 6 years, although these associations seemed largely explained by childhood BMI [8]. Although our current findings need to be interpreted carefully as they are not consistent across GWG exposures, together our current and previous findings might suggests that small cardiac adaptations associated with maternal GWG may track into late childhood, leading to increased left ventricular stroke volume, ejection fraction, and cardiac index during cardiovascular stress. The interpretation for clinical use is difficult, as a u-shaped relationship between left ventricular ejection fraction and higher mortality risk has been reported in adult populations [43]. Further studies are needed to assess the associations of maternal total GWG and excessive GWG with detailed offspring cardiac development from childhood into adulthood, using both detailed cardiac measures for insight into underlying developmental adaptations and clinical cardiac outcomes.The underlying mechanisms involved in the associations between maternal BMI, GWG and offspring cardiovascular health have not been fully elucidated. In addition, to shared environmental, lifestyle-related or genetic characteristics, animal studies strongly suggest direct intra-uterine mechanisms are also involved [2]. Maternal overweight during pregnancy leads to altered maternal and fetal plasma concentrations of glucose, free fatty acids, and amino acids [44], which may adversely affect fetal development. In rodent models, maternal obesity during pregnancy induced cardiac hypertrophy in offspring, accompanied by hyperinsulinemia and activation of insulin signaling pathways [11,12]. Another rodent model observed that offspring from mothers with obesity developed pathologic cardiac hypertrophy associated with re-expression of cardiac fetal genes, causing in young adulthood severe systolic and diastolic dysfunction and cardiac sympathetic dominance [10]. Sympathetic hyper-reactivity is a potentially involved pathway, as offspring of rodents with obesity during pregnancy, showed elevated cardiac and vascular sensitivity to adrenergic agonists. This leads to increased peripheral and coronary vascular resistance during stress, and results in increased cardiac afterload, and enhanced stimulation of cardiac hypertrophy [1,10,32,45,46].

Our results are important from an etiological perspective. This hypothesis generating observational study highlights that in a relatively healthy population maternal pre-pregnancy overweight and high GWG are associated with differences in systolic blood pressure and cardiac response to exercise in adolescent offspring, as compared to normal maternal pre-pregnancy weight and GWG. However, a wider variety of maternal BMI and GWG is needed to investigate if our findings are more pronounced in children of mothers with more extreme weight differences. Although the handgrip exercise is suitable to induce a cardiovascular stress response, it is possible that this response is too marginal to reveal subtle differences in cardiac adaptations. Future studies using different types of exercise, such as treadmill or bicycle exercise, are needed to investigate if the intensity of the exercise may play a role in the detection of cardiac alterations.

### Methodological considerations

Our results align with previous literature and animal experiments, but given our relatively small sample size and the number of tests performed, this study needs to be considered as hypothesis generating. Future studies using larger, more diverse study populations are needed to replicate our findings. Our study population reflects a low-risk population of Dutch nationality only, with a relatively small number of women with extreme underweight or obesity, and a low prevalence of pregnancy complications. This might affect the generalizability of our findings to more diverse, higher-risk populations. CMR is an optimal method to detect subtle differences in the cardiovascular system on a population level, as it is more precise, detailed and less influenced by the performance of the measurements compared to echocardiography. One percent of our CMR scans during exercise were insufficient for assessment and excluded from analysis, mainly due to breath-hold artifacts or problems with ECG triggering [22]. For future research, faster CMR techniques without ECG-triggering or breath-holds could lead to higher image quality during exercise. As in any observational study, residual confounding cannot be excluded. For example, postnatal risk factors, such as diet, physical activity and psychosocial stress may influence adolescent blood pressure. We adjusted our analyses for childhood BMI measured at the time of the MRI visit, as overall proxy for childhood nutritional status, which did not explain observed associations, but information on physical activity and stress were not available. Future studies are necessary to examine the role of these postnatal factors on the cardiovascular stress response. Moreover, we had no statistical power to investigate the influence of potential confounding or effect modification by maternal pregnancy complications, and future research is needed to explore whether similar or stronger effects are present among other, higher‐risk, populations.

## Conclusions

Higher maternal pre-pregnancy BMI and GWG across the full range are associated with higher systolic blood pressure at peak exercise and an altered cardiac response to exercise in offspring aged 16 years, respectively. No consistent associations between maternal pre-pregnancy BMI and left ventricular volumes and function were present. Our results highlight the importance of optimizing maternal weight status before and during pregnancy, to prevent adverse cardiovascular development of the new generation.

**Figure:**
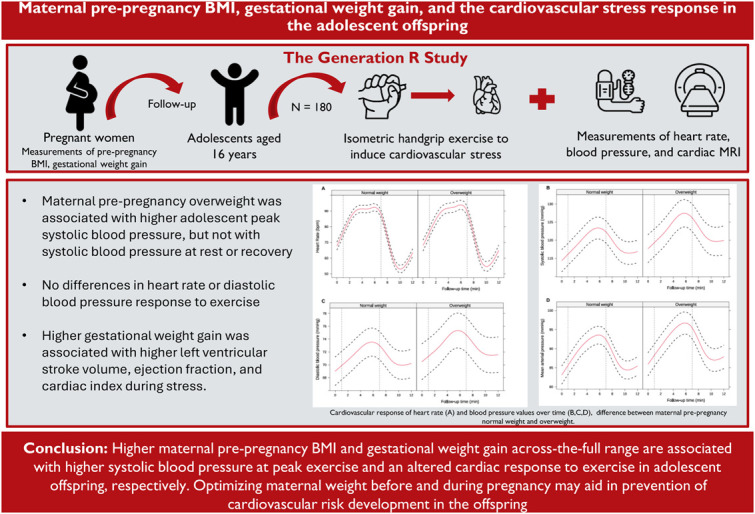
Central illustration.

## Funding

The Generation *R* Study is financially supported by the Erasmus University Medical Centre, Rotterdam, the 10.13039/501100001828Erasmus University Rotterdam, and the Netherlands Organization for Health Research and Development. Vincent Jaddoe received an European Research Council Consolidator Grant (ERC-2014-CoG-648916) and NWO, ZonMW, grant number 05430052110007. Romy Gaillard received funding from the 10.13039/501100003092Dutch Diabetes Foundation (Grant No. 2024.28.001), from the Netherlands Organization for Health Research and Development (NWO, ZonMW, grant number 05430052110007, and NWO, ZonMw VIDI Grant 09150172110034), an 10.13039/501100000781European Research Council Starting Grant (ERC-2024-STG-101161004), and from the European Union’s Horizon 2020 research and innovation programme under the ERA-NET Cofund action (no 727565), EndObesity, ZonMW the Netherlands (no. 529051026).

## Disclosures

A.K, A.R., V.J., R.G. declare they have nothing to disclose. R.B. has nothing to disclose in relation to this article. A.H. has nothing to disclose in relation to this article. In general, A.H. declares that he received a research grant and consultancy fees from GE Healthcare and speaker fees from GE Healthcare, Bayer, Bristol Myers Squibb, Siemens Healthineers, and Heartflow. He is also a member of the medical advisory board of Medis Medical Imaging Systems and was MRI corelab supervisor of Cardialysis BV until 2022.

## Data availability statement

The data that support the findings of this study are available from the corresponding author upon reasonable request.

## CRediT authorship contribution statement

**Arwen S.J. Kamphuis:** Writing – review & editing, Writing – original draft, Methodology, Formal analysis, Conceptualization. **Alexander Hirsch:** Writing – review & editing, Supervision, Methodology, Data curation. **Ricardo P.J. Budde:** Writing – review & editing. **Arno A.W. Roest:** Writing – review & editing. **Vincent W.V. Jaddoe:** Writing – review & editing, Supervision, Funding acquisition. **Romy Gaillard:** Writing – review & editing, Writing – original draft, Supervision, Methodology, Funding acquisition, Data curation, Conceptualization.

## Declaration of competing interest

The authors declare the following financial interests/personal relationships which may be considered as potential competing interests: Alexander Hirsch reports a relationship with GE Healthcare, Bayer, Bristol Myers Squibb, Siemens Healthineers, and Heartflow. member of the medical advisory board of Medis Medical Imaging Systems and was MRI corelab supervisor of Cardialysis BV until 2022 that includes: board membership, consulting or advisory, and funding grants. If there are other authors, they declare that they have no known competing financial interests or personal relationships that could have appeared to influence the work reported in this paper.

## Appendix Supplementary materials

Image, application 1
