# Supplementary material for: Maternal pre-pregnancy BMI, gestational weight gain, and the cardiovascular stress response in the adolescent offspring
Source: Am J Prev Cardiol. 2026 Apr 4;27:101606. doi: 10.1016/j.ajpc.2026.101606 (PMC13261283; doi:10.1016/j.ajpc.2026.101606)
Supplement: Supplementary file 1 [file mmc1.docx]

Supplemental material

**Maternal pre-pregnancy BMI, gestational weight gain, and the cardiovascular stress response in the adolescent offspring**

Arwen S.J. Kamphuis MD^1,2^, Alexander Hirsch MD, PhD^3,4^, Ricardo P.J. Budde MD, PhD^4^, Arno A.W. Roest MD, PhD^5^, Vincent W.V. Jaddoe MD, PhD^1,2^, Romy Gaillard MD, PhD^1,2^

^1^ The Generation R Study Group, Erasmus University Medical Center, Rotterdam, the Netherlands.

2 Department of Pediatrics, Sophia Children’s Hospital, Erasmus University Medical Center, Rotterdam, the Netherlands.

^3^ Department of Cardiology, Cardiovascular Institute, Thorax Center, Erasmus University Medical Center, Rotterdam, The Netherlands.

^4^ Department of Radiology and Nuclear Medicine, Erasmus University Medical Center, Rotterdam, The Netherlands.

^5^ Department of Pediatrics, Leiden University Medical Center, Leiden, the Netherlands.

**Corresponding author**

Gaillard Romy, MD, PhD

The Generation R Study Group (Na-29). Erasmus MC, University Medical Center, PO Box 2040, 3000 CA Rotterdam, the Netherlands. Email: r.gaillard@erasmusmc.nl

**Content:**

Text S1. The cardiovascular stress test protocol

Text S2. CMR protocol

Text S3. Use of linear mixed-effects models

Text S4. Use of Covariates

Figure S5. Directed acyclic graph of maternal pre-pregnancy BMI and gestational weight gain, and the cardiovascular stress response in adolescence

Table S6. Associations of maternal BMI, and gestational weight gain with mean arterial blood pressure at rest, peak exercise and recovery

Table S7. Associations of maternal BMI, and gestational weight gain with aorta distensibility and pulse wave velocity

References

**Text S1 – The cardiovascular stress test protocol**

Briefly, maximal voluntary contraction of the dominant hand was measured using the handgrip dynamometer SS56L hand clench force bulb. During CMR examination, each volunteer was asked to sustain a grasp on the handgrip at 30-40% maximal voluntary contraction for 7 minutes.

**Text S2 – CMR protocol**

The protocol included balanced steady-state free precession (SSFP) cine imaging and 2D phase contrast images. During rest, we acquired localizer images, followed by standard retrospective ECG-gated end expiration breath-held short-axis SSFP scans with coverage from base to apex of the ventricles with a slice thickness of 8 mm. Al breath-holds lasted less than 15 seconds per breath hold. Image analyses were performed by semi-automated analyses, which were manually post-processed by two trained students using dedicated software from Medis Medical Imaging Systems bv Leiden, The Netherlands: Medis Suite (v.3.2.60.6Q), QMass (v.8.1.98.2) and Qflow (v.8.1.98.2). The analyses were performed according to the guidelines of the Society for Cardiovascular Magnetic Resonance (SCMR) ^1^. Both students were trained and closely supervised by a CMR specialized cardiologist (A.H.). We observed good reproducibility for all CMR measurements during rest and stress with coefficients of variation varying from 1 to 11 and a mean intra-class correlation coefficient (ICC) of 0.90 +/- 0.12 ^2^

**Text S3 – Use of linear mixed-effects models**

In the fixed-effects part, we used natural cubic splines with two internal knots placed at the corresponding percentiles of the follow-up times to allow a nonlinear effect of time. We selected the appropriate random-effects structure that best fitted the data based on likelihood ratio tests. The appropriate fixed-effects structure was selected using F and likelihood ratio tests. We used residual plots to validate the models’ assumptions.

**Text S4**: **Use of Covariates**

Potential confounding or mediating variables were selected based on previous literature ^3, 4^, and a directed acyclic graph (Supplementary Figure S5). Given the relatively small sample size, we dichotomized categorized confounders when possible. We used maternal educational level as a proxy for socio-economic status. We dichotomized maternal educational level into low educational level, including no education or primary or secondary education, and higher education. Maternal ethnicity was dichotomized into European and Non-European, according to Statistics Netherlands ^5, 6^. Parity was dichotomized into nulliparous and multiparous. Maternal smoking was categorized in three categories: never smoked during pregnancy, smoked until pregnancy was known, and continued smoking during pregnancy. Maternal age was included as a continuous variable. For the GWG analyses maternal pre-pregnancy BMI was included in the confounder models as a continuous variable. The included potential mediators in the analyses, birth weight, gestational age at birth, and adolescent BMI were included as continuous variables.

**Figure S5**: **Directed acyclic graph of maternal pre-pregnancy BMI and gestational weight gain, and the cardiovascular stress response in adolescence**

**
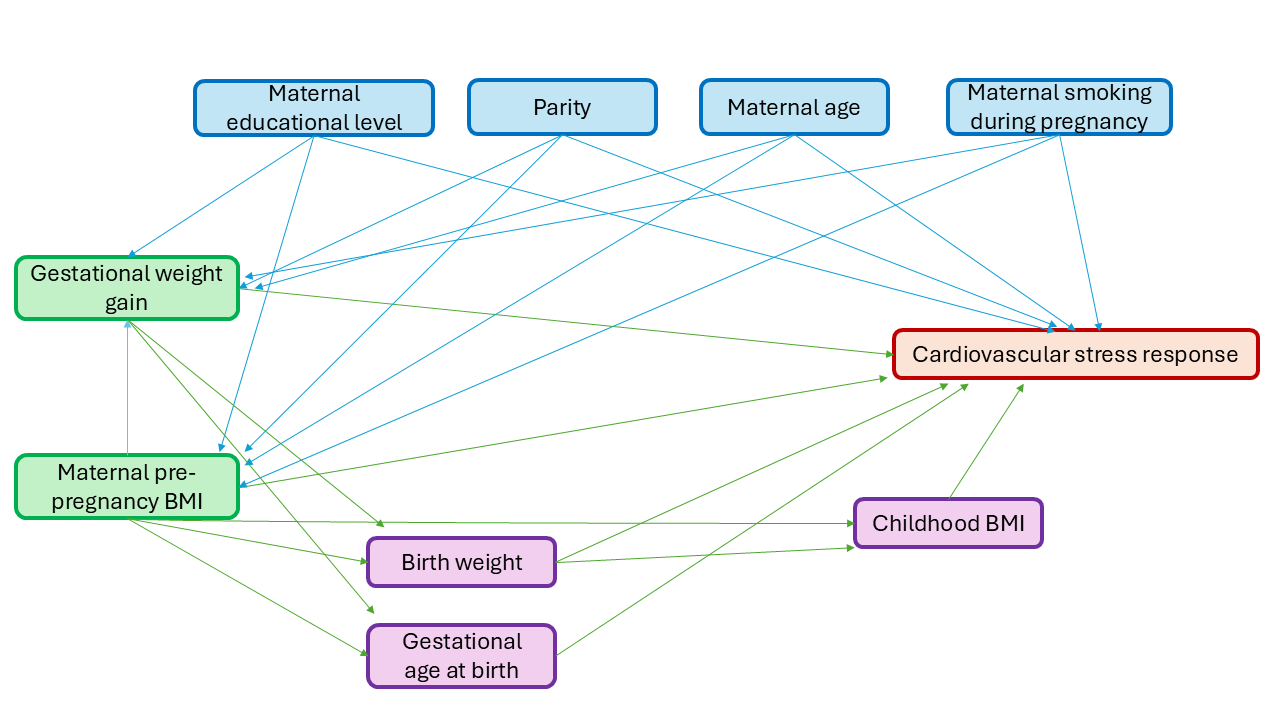
**

Blue arrows**:** potential confounders

Green arrows: potential mediators

| Table S6. Associations of maternal BMI, and gestational weight gain with mean arterial blood pressure at rest, peak exercise and recovery | | | |
| --- | --- | --- | --- |
| Cardiovascular  outcomes: | **MAP**  ***(SDS)***  ***Rest*** | **MAP**  ***(SDS)***  ***Peak*** | **MAP**  **(*SDS)***  ***Recovery*** |
| Basic model |  |  |  |
| Normal weight  *N= 130*  Overweight  *N= 50*  BMI in SDS  *N=180* | Ref  0.45 (0.13 - 0.78)^+^  0.17 (0.03 - 0.31)^+^ | Ref  0.46 (0.14 - 0.78)^+^  0.15 (0.02 - 0.29)^+^ | Ref  0.31 (-0.01 - 0.64)  0.10 (-0.04 - 0.24) |
| Confounder model° |  |  |  |
| Normal weight  *N= 130*  Overweight  *N= 50*  BMI in SDS  *N=180* | Ref  0.36 (0.02 - 0.71)^+^  0.12 (-0.02 - 0.27) | Ref  0.49 (0.15 - 0.82)^+^  0.15 (0.01 - 0.30)* | Ref  0.23 (-0.12 - 0.58)  0.07 (-0.08 - 0.21) |
| Mediator model – Birth characteristics^¶^ | |  |  |
| Normal weight  *N= 130*  Overweight  *N= 50*  BMI in SDS  *N=180* | Ref  0.35 (0.00 - 0.70)^+^  0.12 (-0.03 - 0.27) | Ref  0.47 (0.13 - 0.81)^+^  0.15 (0.00 - 0.30)^+^ |  |
| Mediator model - BMI child^§^ | |  |  |
| Normal weight  *N= 130*  Overweight  *N= 50*  BMI in SDS  *N=180* | Ref  0.26 (-0.10 - 0.63)  0.08 (-0.08 - 0.24) | Ref  0.50 (0.14 - 0.87)^+^  0.15 (-0.00 - 0.30) |  |
| Fully adjusted model |  |  |  |
| Normal weight  *N= 130*  Overweight  *N= 50*  BMI in SDS  *N=180* | Ref  0.27 (-0.10 - 0.64)  0.08 (-0.08 - 0.24) | Ref  0.51 (0.14 - 0.87)^+^  0.15 (-0.00 - 0.31) |  |
| Gestational weight gain – Basic model | |  |  |
| Normal  *N = 95*  Excessive  *N = 85*  Gestational weight gain in SDS  *N = 177* | Ref  0.08 (-0.25 - 0.40)  0.36 (-1.02 - 1.74) | Ref  0.07 (-0.26 - 0.40)  -0.41 (-2.07 - 1.25) | Ref  0.07 (-0.26 - 0.40)  0.38 (-1.12 - 1.88) |
| Gestational weight gain – confounder model° | |  |  |
| Normal  *N = 95*  Excessive  *N = 85*  Gestational weight gain in SDS  N = 177 | Ref  -0.04 (-0.37 - 0.30)  0.01 (-0.15 - 0.18) | Ref  0.00 (-0.33 - 0.34)  -0.07 (-0.23 - 0.09) | Ref  -0.04 (-0.38 - 0.30)  0.01 (-0.16 - 0.17) |
| Values represent regression coefficients (95% confidence interval) from linear regression models that reflect differences in mean arterial blood pressure in mmHg.  °Confounder model: adjusted for maternal smoking during pregnancy, parity, educational level and age  ^¶^Mediator model – birth characteristics: includes all maternal confounders and additionally adjusted for birth weight and gestational age at birth  ^§^Mediator model - BMI child at time CMR: includes all maternal confounders and additionally adjusted for adolescent BMI at MRI  Fully adjusted model: includes all maternal confounders and additionally adjusted for birth characteristics and adolescent BMI at CMR  ^+^P<0.05  BMI: Body Mass Index, MAP: Mean Arterial Pressure, SDS: standard deviation score | | | |

| Table S7. Associations of maternal BMI and gestational weight gain with aorta distensibility and pulse wave velocity | | |
| --- | --- | --- |
|  | **Aorta distensibility**  N = 171 | **Pulse wave velocity**  N = 164 |
| Basic model |  |  |
| Normal weight  *N= 130*  Overweight  *N= 50*  BMI in SDS  *N = 180* | Ref  -0.05 (-0.39 - 0.30)  -0.00 (-0.16 - 0.15) | Ref  0.02 (-0.32 - 0.37)  0.04 (-0.11 - 0.20) |
| Confounder model° |  |  |
| Normal weight  *N= 130*  Overweight  *N= 50*  BMI in SDS  *N = 180* | Ref  0.00 (-0.37 - 0.38)  0.03 (-0.14 - 0.19) | Ref  -0.04 (-0.41 - 0.34)  0.02 (-0.14 - 0.19) |
| Gestational weight gain – basic model | | |
| Normal  *N = 95*  Excessive  *N = 85*  Gestational weight gain in SDS  *N = 177* | Ref  -0.01 (-0.35 - 0.34)  0.01 (-0.16 - 0.18) | Ref  -0.06 (-0.38 - 0.26)  -0.03 (-0.19 - 0.14) |
| Gestational weight gain – confounder model° | | |
| Normal  *N = 95*  Excessive  *N = 85*  Gestational weight gain in SDS  *N = 177* | Ref  0.02 (-0.34 - 0.38)  0.04 (-0.13 - 0.21) | Ref  -0.07 (-0.41 - 0.27)  -0.00 (-0.18 - 0.17) |
| Values represent regression coefficients (95% confidence interval) from linear regression models that reflect differences in BMI (SDS) with large vessel quality in rest. Aorta distensibility in SDS. Pulse wave velocity in LOG-transformed SDS.  °Confounder model: adjusted for maternal smoking during pregnancy, parity, educational level and age  SDS: standard deviation score | | |

**REFERENCES**

1. Schulz-Menger J, Bluemke DA, Bremerich J, Flamm SD, Fogel MA, Friedrich MG *et al.* Standardized image interpretation and post processing in cardiovascular magnetic resonance: Society for Cardiovascular Magnetic Resonance (SCMR) board of trustees task force on standardized post processing. *J Cardiovasc Magn Reson* 2013; **15**(1)**:** 35.

2. Bongers-Karmaoui MN, Hirsch A, Budde RPJ, Roest AAW, Jaddoe VWV, Gaillard R. Physical exercise and cardiovascular response: design and implementation of a pediatric CMR cohort study. *Int J Cardiovasc Imaging* 2023.

3. Gaillard R, Steegers EA, Duijts L, Felix JF, Hofman A, Franco OH, Jaddoe VW. Childhood cardiometabolic outcomes of maternal obesity during pregnancy: the Generation R Study. *Hypertension* 2014; **63**(4)**:** 683-91.

4. Gaillard R, Welten M, Oddy WH, Beilin LJ, Mori TA, Jaddoe VW, Huang RC. Associations of maternal prepregnancy body mass index and gestational weight gain with cardio-metabolic risk factors in adolescent offspring: a prospective cohort study. *Bjog* 2016; **123**(2)**:** 207-16.

5. van Genuchten WJ, Toemen L, Roest AAW, Vernooij MW, Gaillard R, Helbing WA, Jaddoe VWV. Ethnic differences in childhood right and left cardiac structure and function assessed by cardiac magnetic resonance imaging. *Eur J Pediatr* 2021; **180**(4)**:** 1257-1266.

6. Troe EJ, Raat H, Jaddoe VW, Hofman A, Looman CW, Moll HA *et al.* Explaining differences in birthweight between ethnic populations. The Generation R Study. *Bjog* 2007; **114**(12)**:** 1557-65.
